# Analysis of the carbide precipitation and microstructural evolution in HCCI as a function of the heating rate and destabilization temperature

**DOI:** 10.1038/s41598-023-36364-1

**Published:** 2023-06-12

**Authors:** M. Agustina Guitar, U. Pranav Nayak, Lucía Campo Schneider, Jörg Schmauch, Frank Mücklich

**Affiliations:** 1 Department of Materials Science, Saarland University, Campus D3.3, 66123 Saarbrücken, Germany; 2 Department of Experimental Physics, Saarland University, Campus D2.2, 66123 Saarbrücken, Germany

**Keywords:** Materials science, Metals and alloys

## Abstract

Microstructural modification of high chromium cast irons (HCCI) through the precipitation of secondary carbides (SC) during destabilization treatments is essential for improving their tribological response. However, there is not a clear consensus about the first stages of the SC precipitation and how both the heating rate (HR) and destabilization temperature can affect the nucleation and growth of SC. The present work shows the microstructural evolution, with a special focus on the SC precipitation, in a HCCI (26 wt% Cr) during heating up to 800, 900, and 980 °C. It was seen that the HR is the most dominant factor influencing the SC precipitation as well as the matrix transformation in the studied experimental conditions. Finally, this work reports for first time in a systematic manner, the precipitation of SC during heating of the HCCI, providing a further understanding on the early stages of the SC precipitation and the associated microstructural modifications.

## Introduction

High chromium cast irons (HCCI) are abrasion-resistant materials usually employed in applications where a high wear resistance is required, such as mining and mineral processing industries^1,2^. Heat treatments (HT) are employed for microstructure modification after solidification, where secondary carbides (SC) precipitate during the austenite destabilization process^3,4^.

Destabilization of the carbon supersaturated austenite is the most common HT for HCCI. This process allows carbon and chromium to come out of the austenitic matrix by precipitating Cr-rich SC. The carbon depletion from the austenite results in an increase of the martensite start temperature (Ms), allowing the HCCI to be hardened by both carbide precipitation, and martensite formation^4–9^ during quenching. Usually, the destabilization (also called critical) process takes place at temperatures range between 800 and 1150 °C and holding times up to 8 h for maximum hardening of the alloy^2,3,9–12^. The type of SC formed during destabilization depends on several factors, including the destabilization temperature, the alloy composition (especially Cr/C ratio), and the destabilization holding time. Precipitation of M_7_C_3_ is expected for alloys with Cr/C < 6.8^1,3^, whereas for alloys with higher Cr content (> 25 wt% Cr; Cr/C > 6.8) the precipitated carbides are of the M_23_C_6_ type^1,9,10^. However, despite the alloy composition, for which M_7_C_3_ are predicted to be thermodynamically stable, Powell observed the preferential formation of M_23_C_6_ type SC in an alloy with Cr/C ratio of 5.7, owing to the better austenite/M_23_C_6_ lattice match^10^. It was also observed that M_7_C_3_ was present after 4 h of destabilization at 1000 °C, but it was uncertain whether the M_23_C_6_/M_7_C_3_ in-situ transformation was primarily due to internal diffusion process.

Nevertheless, it is not possible to find a clear consensus concerning the initial stages of carbide precipitation. Most of the studies dealing with the kinetics of SC precipitation agreed that the precipitation occurs actually during the first 5–30 min of the holding period, depending on the employed temperature, as a result of re-ordering of the carbon within the austenitic matrix^10,11,13^. After that, only growth and coalescence of carbide can occur. Efremenko et al. observed the starting of the precipitation process after an incubation period of 10 s, independent of the alloy´s composition studied^13^. Furthermore, carbide precipitation during cooling was also mentioned in the review published by Tabrett, especially in the temperature range 800–900 °C^1^. This phenomena was also suggested by Wang^14^ after detecting very fine M_23_C_6_ SC after quenching to a cryogenic temperature. Additionally, in an own previous work^4^, thermodynamic and kinetic simulations suggested that the SC precipitation starts already during the heating process at temperatures around 800 °C. During this stage, M_7_C_3_ carbides precipitate initially, and eventually give way to the precipitation of M_23_C_6_ carbides once the destabilization temperature of 980 °C is attained. However, from the simulations alone it is not possible to inquire whether the M_7_C_3_ carbides dissolve or transform, by a diffusion process, into M_23_C_6_. Additionally, the simulations also suggested that during the first stage of the cooling further precipitation can take place, as mentioned by Wang and Tabrett^1,14^.

Based on the previous, it is seen that there are still some controversies about the exact occurrence of the SC precipitation. This is combined with a poor understanding and a lack of information about the SC formation during heating as well as the effect of heating rate on the precipitation process. For these reasons, the purpose of the present work is to study the microstructural evolution, especially of the SC, in a HCCI (26 wt% Cr) during heating up to different temperatures. Additionally, the effect of the heating rate (HR) in the carbide precipitation as well as in the matrix transformation are also analysed and discussed here. Furthermore, the presence of M_7_C_3_ SC precipitating during heating (as suggested by the simulations performed by Guitar et al.^4^) will be evaluated by implementing transmission electron microscopy (TEM) and atom probe tomography (APT) analysis.

## Materials and methodology

For this work, 20 mm × 20 mm × 10 mm samples of a HCCI alloy containing 2.53 wt% C, 26.6 wt% Cr and other minor alloy elements^15^ were heat treated up to three different temperatures (800, 900, and 980 °C) using two different HR, 1 and 10 °C/min. The nomenclature corresponding to each of the samples is shown in Table 1. Once the target temperature was reached, the samples were taken from the oven and immediately water quenched to retain the microstructure. The as-cast material consisted of M_7_C_3_ eutectic carbides (EC), an austenitic matrix and a martensitic layer at the matrix/EC interface, as previously described^4,7,15^.The treatment temperatures were selected based on previously published and unpublished results obtained from three different methods namely: high-temperature X-ray diffraction, dilatometry, and MatCalc kinetic simulations^4^, and the temperatures typically employed for the destabilization of HCCI^4,16^, where the temperature range 950–1000 °C corresponds to the highest precipitation rate^13^.

**Table 1:** Nomenclature of the heated samples at the different HR, up to the three different temperatures.

HR	T		
	800 °C	900 °C	980 °C
1 °C/min	800_1	900_1	980_1
10 °C/min	800_10	900_10	980_10

Phase identification was performed by X-ray diffraction using a PANalytical Empyrean diffractometer system equipped with a Bragg Brentano-HD (BBHD) module and an ultra-fast PIXcel-3D detector. A symmetrical θ-2θ scan geometry and Cobalt (Co) (Kα = 0.1791 nm) radiation source were used. The applied acceleration voltage and current were 40 kV and 40 mA, respectively. The scan range was 40–130° with a step size of 0.013° and a counting time of 250 s. Moreover, the pulse height distribution (PHD) settings were set to a range of 25.5% (3.53 keV)–80% (11.09 keV). The X’Pert High Score Plus software and ICDD Database were used for peak indexing.

Post HT, the samples were ground and polished following the procedure described in^17,18^ to obtain a scratch free, mirror polished surface. For general microstructure revelation, the samples heated up to 800 °C were etched with Villella´s reagent (1 g picric acid + 5mLHCl + 95mLC_2_H_5_OH)^8,17^, whereas samples heated up to 900 and 980 °C were etched with Nital (98 mL ethanol + 2 mL nitric acid + 0.5 mL HCl)^17^. The microstructure was analysed using a FEI Helios Nanolab field emission scanning electron microscope (FE-SEM) working with an acceleration voltage of 5–15 kV and a beam current of 1.4 nA. Electron backscattered diffraction (EBSD) was used to investigate the distribution of the microstructural constituents, especially the retained austenite (RA). The measurements were performed at an acceleration voltage of 20 kV and a beam current of 11 nA. The EBSD data was analysed using the Orientation Imaging Microscopy (OIM™ v. 7) Data Analysis software by EDAX Inc.

For the determination of the size and volume fraction of the SC, the samples were etched with a modified Murakami’s reagent (4 g K_3_[Fe(CN)_6_] + 8 g NaOH + 100 mL H_2_O) at room temperature for 15 s and analysed with the FE-SEM using a high sensitivity backscattered electron detector (vCD) for a better contrast between the phases, as shown^17^. The carbide volume fraction (CVF) and the SC size were calculated after a post processing of the images using the image analysis (I-A) software, ImageJ (version 1.52p)^19^. The same area was evaluated in all the images and at least 5 micrographs were processed in each case^20^. Only particles composed for at least 2 pixels were included in the analysis, i.e., considering the magnification (2500x) and resolution (4088 pixel × 3523 pixel) of the image, all pixelated particles having a diameter less than 30 nm were disregarded.

**Figure 1:**
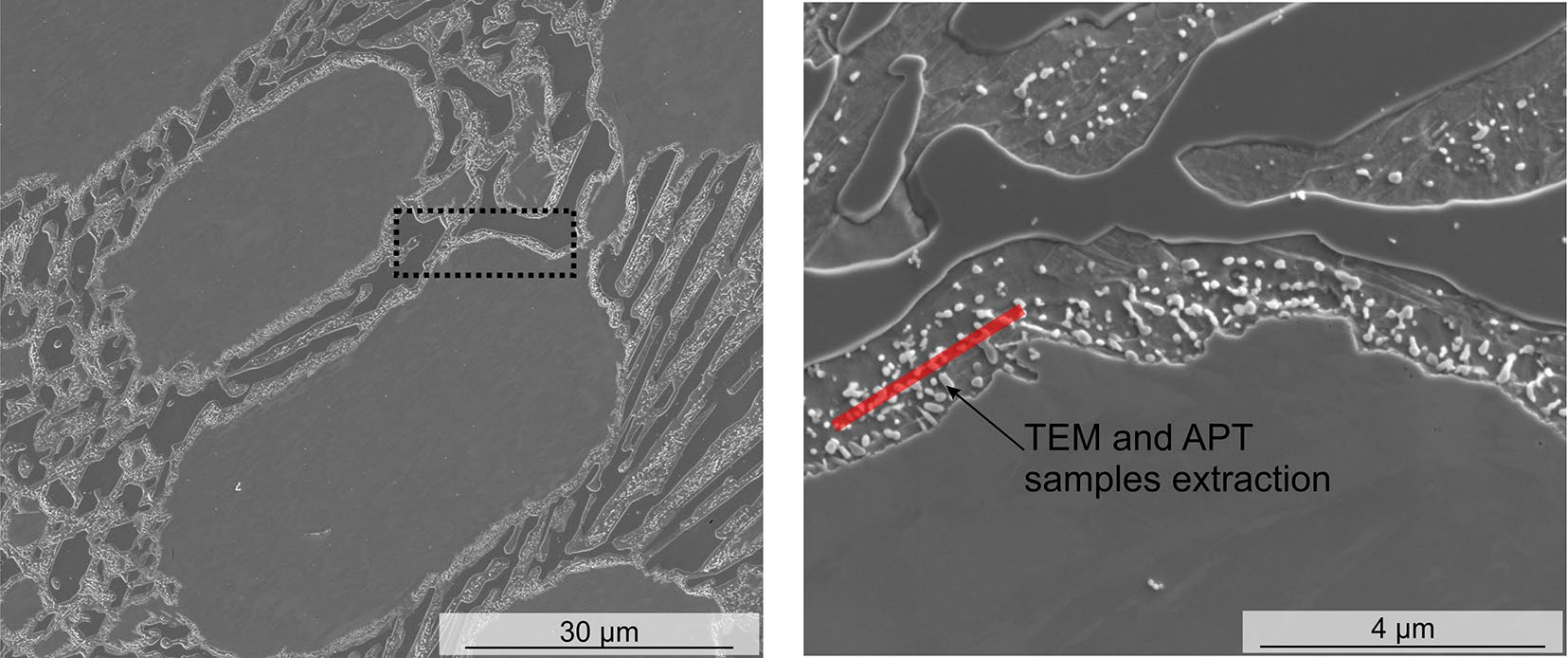
Representative extraction site for the TEM lamella and APT sample. For the analysis the 800_10 sample was used.

For the identification of the SC, TEM and APT samples were extracted from the regions next to the EC, where a higher density of SC was observed (Fig. 1). For that, measurements with high-resolution transmission electron microscopy (HR-TEM) using a JEOL ARM 200 TEM/STEM equipped with a Cs corrector (CEOS GmbH), and atom probe tomography (APT) in a LEAP™ 3000 HR (CAMECA Instruments, Madison, Wi, USA) were performed. The APT measurements were carried out in voltage mode, pulse frequency 200 kHz, voltage pulse 20% of standing voltage and evaporation target of 1 event per 200 pulses. Different measurements at temperatures between 60 and 70 K were performed to evaluate the effect of changing the temperature in the measured carbide composition. Finally, the best result which was obtained at 60 K is presented here. Further information about the results at the different measurement temperatures can be found in the supplementary material. The TEM samples as well as the needle shaped specimens for APT were prepared with focus ion beam (FIB, FEI Helios Nanolab 600, FEI Company) as detailed in reference^21^ and^22^, respectively. A final polishing with 5 kV (TEM samples) and 2 kV (APT samples) was performed in order to decrease the Ga contamination.

The 3D reconstruction and the peak decomposition from atom probe data was performed using the software IVAS™ 3.6.14 (CAMECA Instruments, Madison, Wi, USA), applying tip profile algorithm. For that, SEM images of the tip before APT measurement were used. The peak decomposition results were exported for further calculations to MATLAB R2020b. The mass spectrums images and the multiple hit analysis were performed using APT toolbox for MATLAB^23^. Furthermore, the carbides were indexed on Fast Fourier Transform (FFT) images from HR-TEM micrographs, which contains similar information to electron diffraction patterns when a single crystal is analysed. For SC identification the CrysTBox software^24^ was used.

## Results and discussion

**Figure 2:**
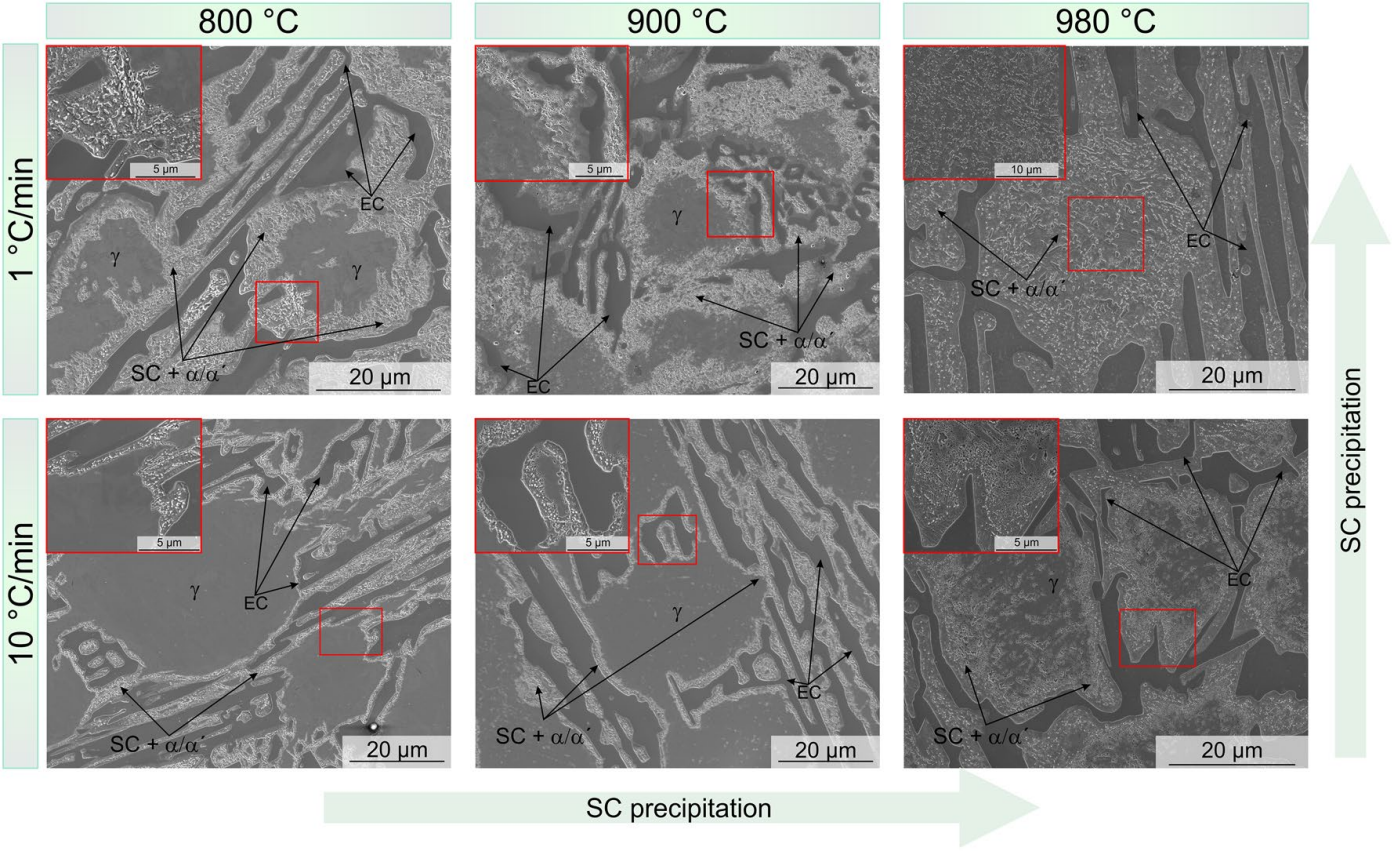
SEM images corresponding to the samples heated under the different conditions.

The microstructure of the heat-treated samples after reaching the desired temperature and using two different HR is shown in Fig. 2. The presence of SC can be observed even after heating up to a relatively low temperature (800 °C), which is an indication that precipitation occurs already during heating, starting at temperatures below 800 °C. In the literature, it is not possible to find a concurrence about the start of the carbide precipitation. Many authors report the presence of SC after a few minutes of holding at the destabilization temperature. Efremenko et al.^13^ is one of the few suggesting that precipitation starts after soaking for only 10 s at 950 °C in HCCI with different compositions. Still, there is no mention or proof of precipitation during heating. Therefore, the results shown here represent the first experimental evidence of SC precipitating during heating, starting at temperatures below 800 °C. This is in agreement with the results previously shown in^4^, where kinetic simulations predicted the precipitation of SC during this step of the HT.

**Figure 3:**
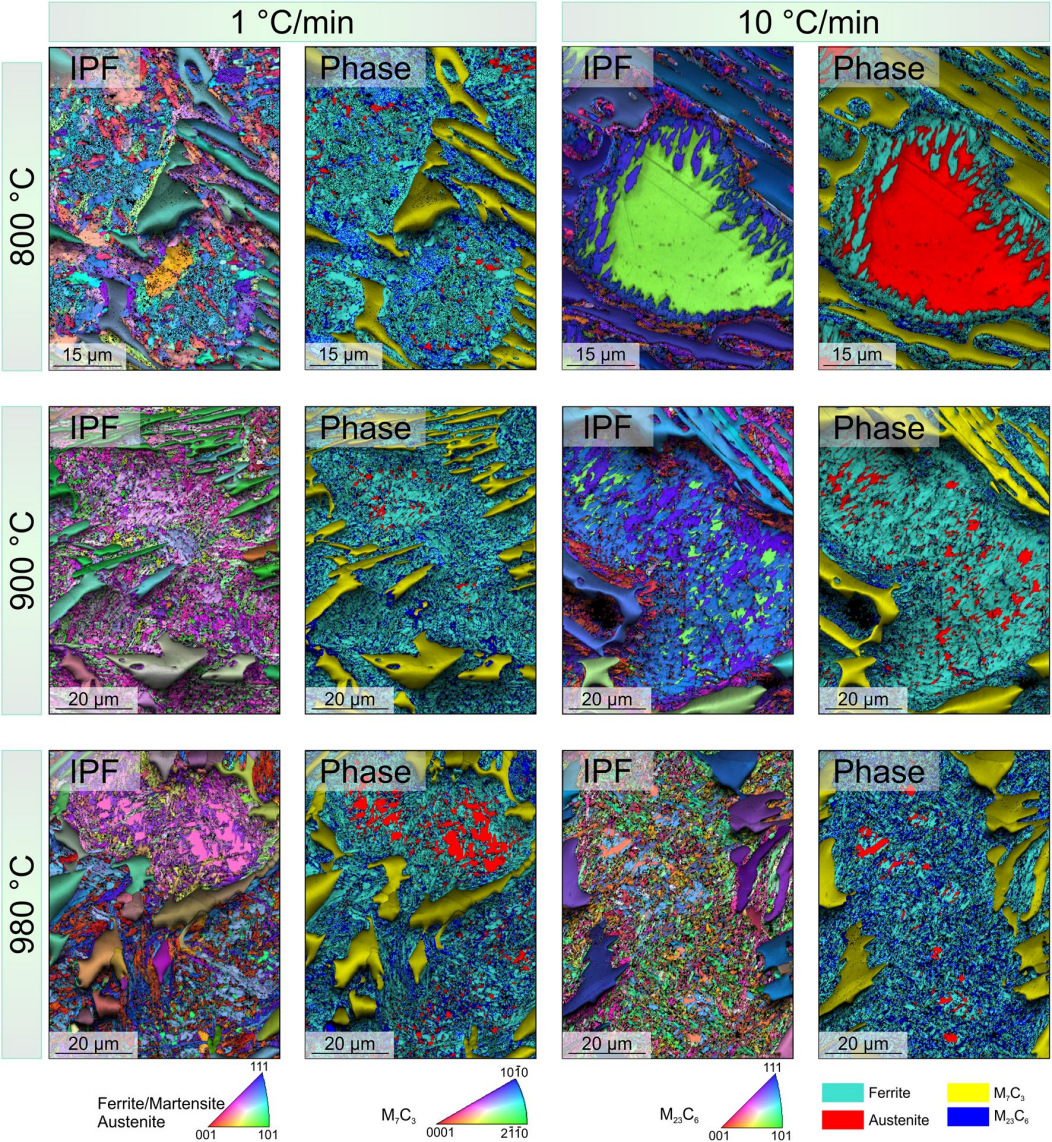
IPF + IQ and Phase + IQ maps corresponding to the samples heated at two heating rates up to the three analysed destabilization temperature. Larger transformed areas are observed in samples slow heated. Consider that EBSD maps might fail in the quantification of RA, due to low statistic and possible austenite to martensite transformation during preparation.

**Figure 4:**
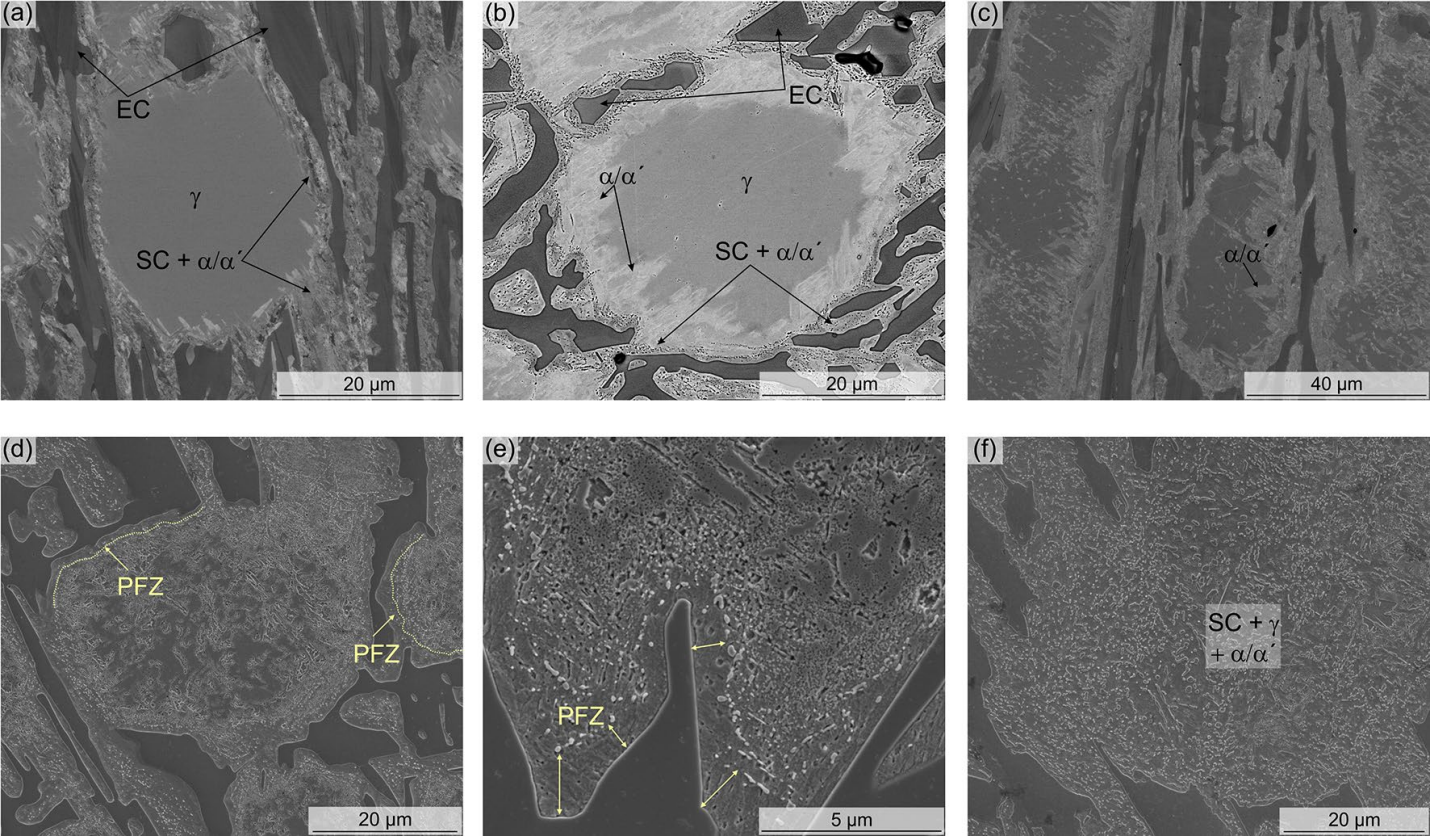
SEM images corresponding to the (**a**) 800_10 sample, polished with OPS; (**b**) 800_10 sample (Murakami etched) in VcD mode; (**c**) 900_10 sample, polished with OPS showing regions of martensite/ferrite free of visible carbides; (**d**) and (**e**) 980_10 sample showing precipitate free zone; and (**f**) 980_1 sample showing the martensite/ferrite matrix covered by SC.

The SC start to precipitate at the regions next to the EC, evidenced by the relatively high density of small particles present there, where two clear trends can be seen in the microstructural evolution of the heated samples in Fig. 2: (i) SC precipitation increases with temperature, (ii) SC precipitation increases with decreasing of HR. Both the increase of the reached temperature and the decreasing HR, led to SC precipitation towards the centre of the austenite grains (Fig. 2). About twice the area is covered by SC in the 800 and 900 °C samples heated at 1 °C/min compared to those heated faster. Moreover, the re-distribution of alloy elements resulted in a partial transformation of austenite to martensite during cooling, even in regions free of detectable SC, as seen in EBSD maps (Fig. 3) and BSE-SEM images (Fig. 4). Karantzalis et al. reported that experiments carried out at 750 °C showed a partial transformation from austenite to ferrite and pearlitic-like structures whereas, the hardness increase reported after HT at 850 °C was related to the presence of martensite and SC^12^. Based on that, the microstructure of samples 800_1 and 800_10 may consist of both ferrite and martensite due to the temperature of 800 °C utilized in the study, which is on the threshold between the “destabilization temperature” (referred to as the critical temperature) and “subcritical temperature^12^. Since the martensite cannot be straightforwardly separated from the ferrite using the characterization techniques employed here, throughout the text martensite/ferrite phase will be utilized, when referring to the heat-treated conditions. Finally, Fig. 3 (800_10) and Fig. 4a, b) show SC located at the vicinity of the EC however, the martensite/ferrite phase extends few microns along the matrix. Slower heating rates allow the homogenization of alloy elements distribution, leading to nucleation and growth of SC across a larger area of the matrix, where about 17% of the sample is not covered by SC in the 800_1 sample compared to about 35% of carbide-free area in the 800_10 sample.

The martensitic region at EC/austenite interface (as-cast condition)^15^ possesses a large lattice distortion and non-equilibrium defects^25^, resulting in a reduction of the activation energy required for nucleation of small-sized carbides^11,13^. As a consequence, the carbide precipitation is favoured at the vicinity of the EC (Fig. 2, samples 800_10 and 900_10). Moreover, the carbon supersaturation of the martensitic phase combined with the faster diffusion coefficient also benefit the formation of SC on defect sites such as dislocations or lath boundaries^26,27^.Some authors suggested that the M_7_C_3_ EC might act as C reservoir, allowing the precipitation of SC by releasing C and increasing locally the amount of C^9,28^. The release of C leads to a partial transformation of the EC periphery from M_7_C_3_ to M_23_C_6_. The proposed mechanism seems not be the one acting for the beginning of precipitation in the samples studied here, since it was not possible to detect any M_7_C_3_ to M_23_C_6_ transformation at the periphery of the EC (Figs. 2 and 3).

Other authors^28^ made reference to a precipitates free zone (PFZ) located at the primary carbides/austenite interface after long holding times (< 16 h). They suggested that the PFZ is the consequence of Cr solubility/concentration for SC precipitation not being reached at this region, concluding that PFZ will disappear without a Cr gradient. In the material studied in the present work, the martensitic region at the EC/austenite interface is the consequence of a Cr and C gradient. However, the only sample showing a PFZ is the one heated up to 980 °C with the faster HR (Fig. 4d, e). Nonetheless, the differences in the treatment conditions and C content between Roussel´s work and the present work should be taken into consideration. However, it is of interest to evaluate the kinetic conditions leading to the precipitation of carbides, where the conditions, from the point of view of chemical gradients, are not the most optimum. On one hand, the martensitic region at the EC/austenite interface is supersaturated in C and contains a large number of defects, as discussed previously. With an increase of the temperature, the mobility of C in martensite increases rapidly^26^. On the other hand, due to a less availability of Cr in this region, the nucleation of low Cr carbides such as (Cr,Fe)_7_C_3_ might be possible, as suggested by previous kinetic simulations reported in^4^.

An increase in the temperature from 800 to 900 °C (HR 10 °C/min) led to additional SC precipitation along dendritic interfaces, as consequence of further alloy elements re-distribution. As seen in the sample 900_10 (Fig. 4c), small precipitates have nucleated at dislocations or dendrite boundaries within the austenitic grain. The preferential SC precipitation at these sites is again related to a higher local density of defects^29^. Furthermore, for the same reached temperature and slower HR, the time provided for redistribution of alloy elements was enough for more massive carbide precipitation, evidenced by the larger area transformed during the process as shown in Fig. 2 (samples 800_1 and 900_1). Finally, with an increase of temperature of only 80 °C, massive carbide precipitation can also be observed also within of the austenitic region (Fig. 2), for both conditions HR 1 °C/min and 10 °C/min. This corresponds to the observations in other works, where it was seen that that the SC precipitation rate was the highest between 950 and 1000 °C^9,13^.

**Figure 5:**
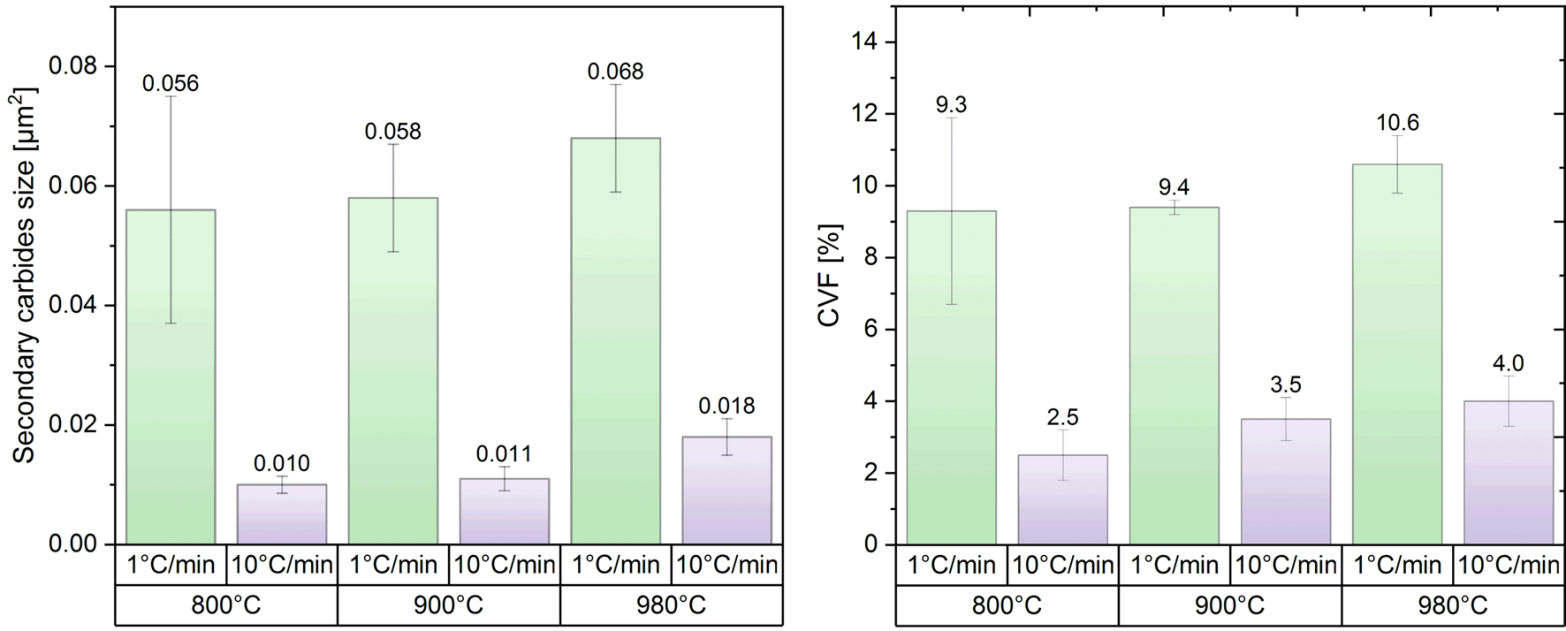
Size and volume fraction of the secondary carbides precipitated during heating, as a function of heating rate and reached temperature.

The calculated secondary carbide volume fraction (CVF) and the corresponding average size are represented in Fig. 5. There, a strong effect of the HR can be seen in both the particle size and secondary CVF. Although a slight tendency to larger size and CVF values can be distinguished with increasing temperature for a certain HR, they remain within the tolerance interval. An increment in the temperature from 800 to 980 °C led to an increase of about 30% in the particle size and about 14% in the CVF. On the other hand, for the fastest HR, the carbides doubled in size and CVF increased by 60% when reaching 800 and 980 °C, respectively. Moreover, a slow HR results in carbides size up to 5 times increase, whereas the CVF increased by about 3.5 times compared to the faster HR for the same reached temperature. From here, it is clear that the main factor affecting the SC size and CVF is the HR and to a lesser extent the reached temperature, as seen from Fig. 5.

The reached temperature, as well as the HR, affect both the fraction and size of the SC, and the fraction of RA, since it is directly related to the C remaining for austenite stabilization. Austenite can be stabilized and retained using different routes. One is performing destabilization at high temperatures (above 1000 °C)^30,11^, destabilizing for very short times (less than 5 min)^16^, or performing the destabilization at low temperatures (under 900 °C). The result, in terms of material´s response can be very different^5^. Furthermore, the different microstructural configurations resulting from varying the temperature and HR affect the tribological response of the material, as described by Nayak et.al.^31^. There, it was demonstrated that the slow HR samples, despite showing an increased SC CVF and size and higher hardness, they showed a lower wear resistance than the faster HR samples. This evidenced the relevance of studying the effect of HR on the SC precipitation and the subsequent microstructural evolution.

**Figure 6:**
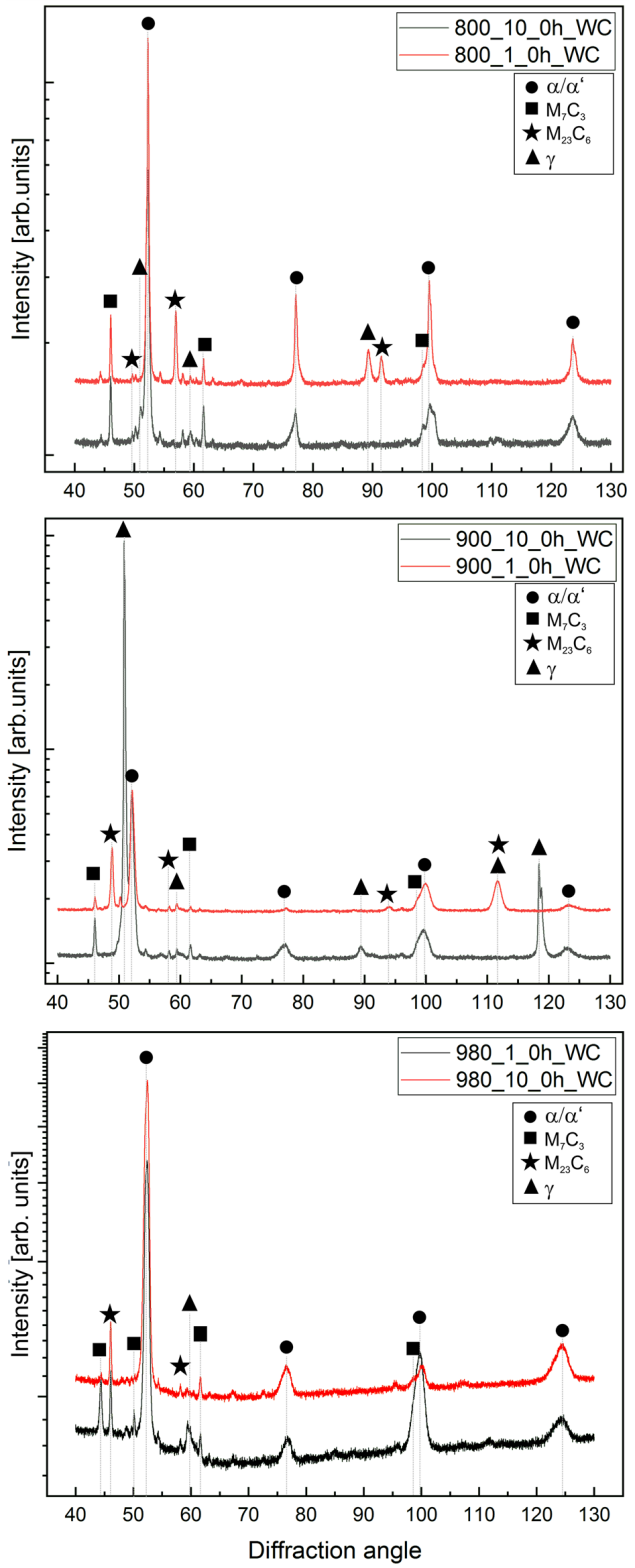
Diffractograms of the samples heat treated samples as a function of the heating rate for the different temperatures analysed. The intensity is shown in logarithmic scale for better comparison. Peaks corresponding to martensite and ferrite appear usually in almost same position and they cannot easily be separated, therefore the peaks were indexed as α/α′.

Figure 6 displays the diffractograms of the different heat-treated samples, showing the presence of M_7_C_3_ and M_23_C_6_ carbides, austenite, and martensite. Peaks of M_23_C_6_ carbides correspond to the SC precipitated during heating, which can be observed in the samples heated at 1 °C/min and less intensely in the samples heated faster. Interesting is the case of the samples heated up to 800 and 900 °C at 10 °C/min, which apparently show same microstructural distribution and fraction of SC, show different fraction and distribution of RA (see Fig. 3). In the latter case (900_10 sample), XRD shows an intense peak of RA, suggesting an additional process occurs during heating up to 900 °C related to alloy element redistribution, probably the formation of a few nm size carbide nucleus, small enough not to be detected by SEM. Even though it is expected that the samples heated up to 800 °C contain the largest fraction of RA, they show the less intense peak of austenite, which is probably related to a sampling effect of the large austenitic grains^32^. Even though EBSD maps in Fig. 3 might not be representative enough, it shows a relatively good idea of RA austenite distribution. In all cases, M_7_C_3_ peaks correspond to the EC, which do not show any visible modification, in terms of size and shape, during heat treatment such as refinement or fractioning. The potential presence of SC in form of M_7_C_3_ cannot be visualized by X-ray diffraction, due to the low fraction expected^4^, which would not significantly alter the intensity of M_7_C_3_ peaks from the EC.

**Figure 7:**
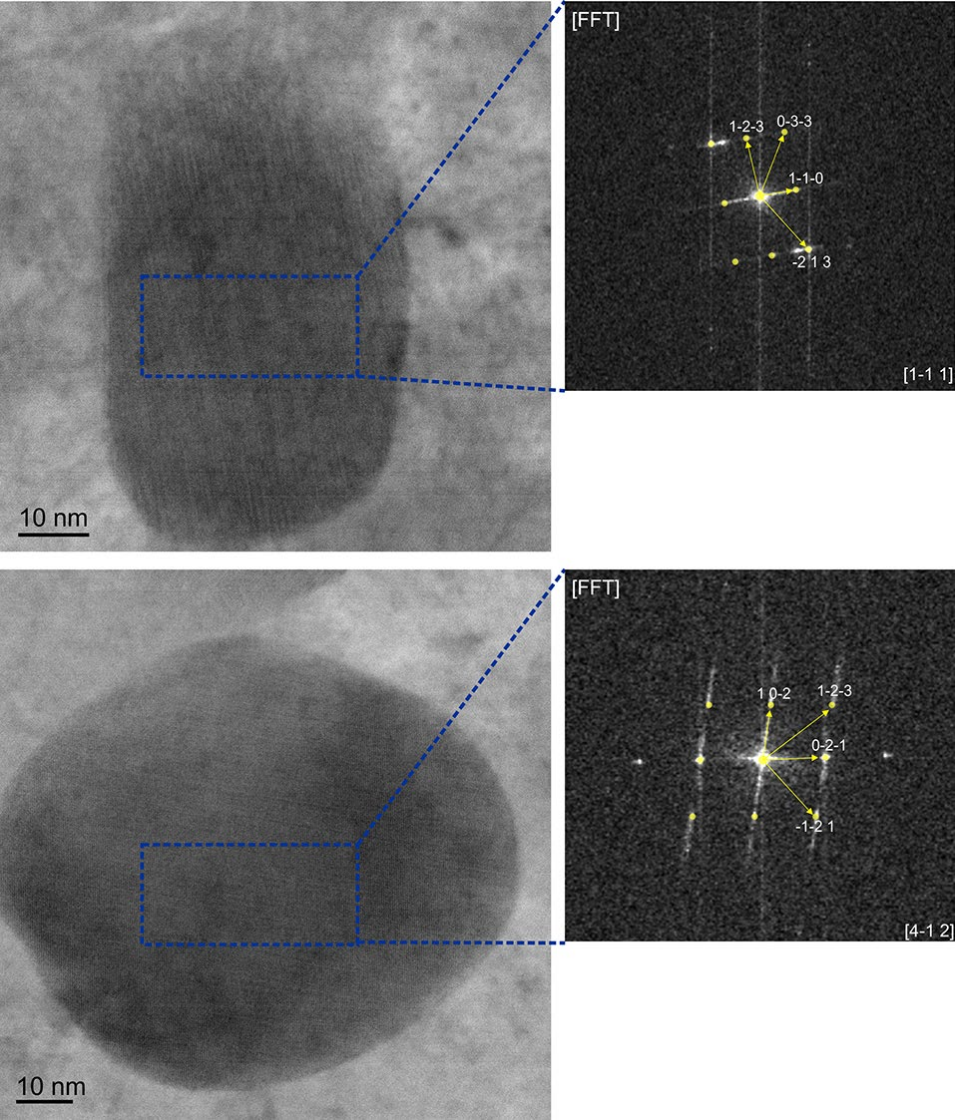
HR-TEM images of two different particles extracted from the 800_10_0h sample, which were identified as M7C3 carbides with help of the CrysTBox software^24^. The corresponding Fourier transformations are shown.

As mentioned previously, kinetic simulations and lower availability of Cr in the region next to the EC suggest the possibility of precipitation of (Cr,Fe)_7_C_3_ type SC. Due to their size and volume fraction, their detection with XRD is not viable. Therefore, TEM samples were extracted from the region next to the EC (Fig. 1), where the beginning of SC precipitation was observed The particles, with sizes of around 50–60 nm in diameter (Fig. 7), were indexed by the CrysTBox^24^ as M_7_C_3_.

**Figure 8:**
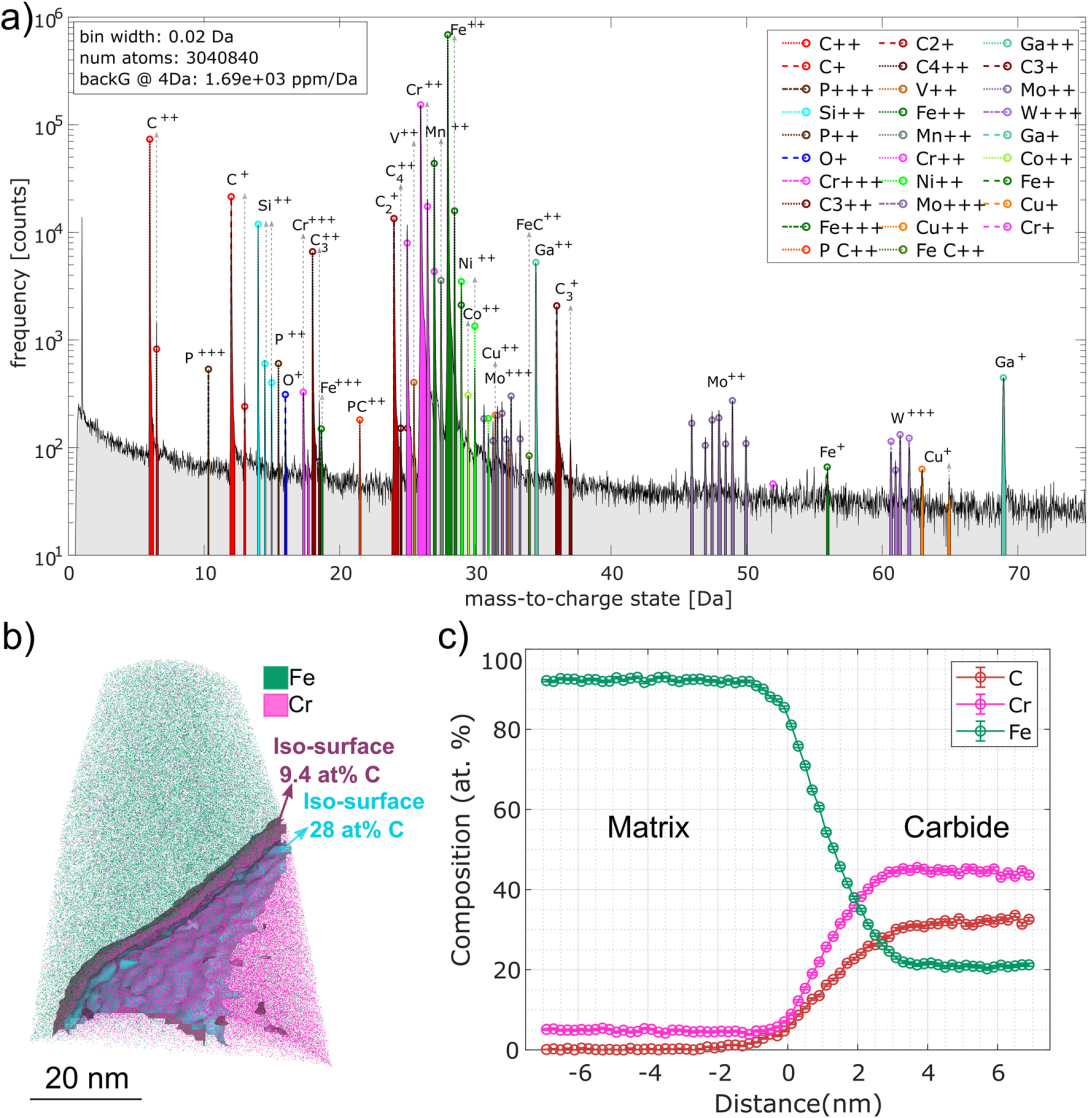
(**a**) Mass spectrum of the specimen presented in (**b**), measured at 60 K. The occurrence of different ions of single and molecular C is evidenced and the complexity of the spectrum due to overlapping of several peaks. The presence of Ga ions is the result of the FIB preparation. (**b**) Reconstruction of and specimen containing part of a carbide. For clarity Fe and Cr atom are shown. An iso-surface of C = 9.3 at.% was created to calculate the variation of composition across the interface. The second iso-surface at 28 at.% was utilized to define a ROI where to perform the compositional analysis. (**c**) Proxigram across the iso-surface at 9.3 at.% showing that after 2–3 nm the C content inside the carbide tends to 30 at.%. Please note that no peak decomposition is performed for the proxigram.

Moreover, APT samples extracted from a similar region as the TEM samples, were analysed for determination of the chemical composition of the particles, as shown in Fig. 8. The reconstruction of a specimen containing part of a carbide can be seen in Fig. 8b, where the Fe and Cr atom are shown. A ROI including only the carbide volume (delimited by the 28 at.% iso-surface) was exported and analysed for determining the composition of the carbide. Regions close to the interface were avoided to minimize error generated by ion trajectory overlap that can occur due to the presence of phases with different evaporation field^33^.

As a result of the peak decomposition analysis, a C content of 29 ± 1 at.% is obtained (Fig. 8c), which is very close to the 30 at.% C expected for M_7_C_3_ carbide. It is important to remark the challenges associated to the quantification of carbon-rich phases resulting from the over-^34–36^ and underestimation^37–39^ of carbon due to overlapping of molecular carbon preferential evaporation and pile up effect. However, the results reported by Takahashi et al.^36^, and Marceau et al.^34^, showed that for the working temperature range (70 °K) the 24 Da is mainly C_2_^+^ (Fig. 8a) and the peak decomposition algorithm provides a reliable result. A more detailed analysis of these effects and its impact in the carbon quantifications is provided in the supplementary material. Although the proxigram in Fig. 8c) only shows the content of C, Cr, and Fe, all elements labelled in Fig. 8a) were included for the compositional analysis of the carbide. The individual element quantification can be seen in the supplementary material.

Results shown from HR-TEM and APT support the simulations performed in a previous work^4^ that SC of the M_7_C_3_ nature start to precipitate during the heating of HCCI. It is also worth to note that the presence of M23C6 type SC was also detected during HR-TEM analysis, which was previously confirmed in samples of the same composition^4^. Powell^40^ gave some indication of possible precipitation of M_7_C_3_ together with M_23_C_6_ carbides in HCCI 18% Cr when destabilizing for short times (0.25 h) at 1000 °C. It is known that M_7_C_3_ can be predecessor of M_23_C_6_, as well as M_23_C_6_ can be predecessor of M_7_C_3_, highly depending on the bulk local chemical composition^41^. Moreover, both type of carbides were seen after destabilizing for longer times, where also a partial transformation from M_23_C_6_ to M_7_C_3_ was observed^9,42,43^. In the current work, the presence of both M_7_C_3_ and M_23_C_6_ type of carbides were detected at the beginning of the destabilization process, i.e., during heating. However, from the results shown here, it is not completely clear whether the precipitates nucleating first as M_7_C_3_, with a (Cr_4.7_Fe_2.3_)C_3_ composition, transform later to M_23_C_6_ or they dissolve giving place for the precipitation and growth of M_23_C_6_.

## Conclusions

The influence of the HR and the reached destabilization temperature on the SC precipitation in HCCI during heating was evaluated. The results presented here provide an understanding on the early stages of the SC precipitation and the associated microstructural modifications. Even though other techniques (e.g., DSC) might provide further information about the exact temperature of the onset of carbide precipitation, the present work provides a description of the first stages of carbide precipitation that are well supported by the extensive microstructural analysis and characterization employing complementary characterization techniques that cover a wide range from the macro scale (XRD), to the micro- and nano- scale (SEM, EBSD, HR-TEM) down to the atomic scale (APT).

The microstructural modification of HCCI containing 26 wt% Cr starts during heating with the precipitation of SC at temperatures around 800 °C. It is accompanied with a redistribution of alloy elements, which lead to the matrix transformation to α/α′ and allows the retention of different fraction of RA. The morphology, fraction, and distribution of the phases within the material depends on both the HR and the reached temperature. These two parameters influence the size and VF of the carbides precipitated during heating. Despite a tendency towards an increased carbide size and CVF when the temperature is increased from 800 to 980 °C, it is clear that the HR has the strongest influence on the carbide volume fraction and particle size reached after heating. A slow heating rate provides longer time at higher temperatures, allowing the nucleated particles to grow and coalesce during the heating period.

Finally, this work reports for first time, in a systematic manner, the precipitation of SC during heating of the HCCI, where the HR was the most dominant factor in the size and volume fraction of the SC present at different heating stages. Through crystallographic and chemical analysis, it was possible to support the theoretical aspects related to the precipitation of both type of carbides, M_7_C_3_ and M_23_C_6_ during the heating process, where M_7_C_3_ SC possess a (Cr_4.7_F_2.3_)C_3_ composition as shown by APT results.

## Supplementary Information

Supplementary Information.

## Data Availability

The data and materials used in this study are available from the corresponding author at reasonable request.
