# Supplementary material for: Analysis of the carbide precipitation and microstructural evolution in HCCI as a function of the heating rate and destabilization temperature
Source: Sci Rep. 2023 Jun 12;13:9549. doi: 10.1038/s41598-023-36364-1 (PMC10261120; doi:10.1038/s41598-023-36364-1)
Supplement: Supplementary file 1 — Supplementary Information. [file 41598_2023_36364_MOESM1_ESM.docx]

**Analysis of the carbide precipitation and microstructural evolution in HCCI as a function of the heating rate and destabilization temperature**

M. Agustina Guitar

U. Pranav Nayak

Lucía Campo Schneider

Jörg Schmauch

Frank Mücklich

**Supplementary material**

The composition of secondary carbides (SC) precipitated during the heating step was determined by Atom Probe Tomography (APT). The objective was to confirm the presence of M_7_C_3_ type SC at the initial stages of the precipitation. The analysis was performed on the sampled heated until 800ºC (HR 10 °C/min) and quenched, form regions close to EC, as shown in Figure 1 of the main text.

A total of 6 specimens were measured, from which 4 included parts of carbides. Here the analysis of 3 carbides are shown, measured in three different temperature conditions between 60 and 70 K. For the carbide compositional determination, a region of interest (ROI) including only the carbide volume was exported and analyzed. Regions close to the interface were avoided in order to minimize any error generated by ion trajectory overlap resulted from the presence of phases with different evaporation field ^1^.

Figure S1 shows the mass spectrum of one of the samples, measured at 70 K. There, it is seen that C is detected as C^++^, C^+^, C_3_^++^, C_2_^+^, C_4_^++^, C_3_^+^. The overlap between the different peaks is one of the issues affecting the carbon quantification. For example, the peak at 24 Da can be assigned to C_2_^+^ or C_4_^++^ (which would contribute to twice amount of C in that peak). As known from the literature, this peak is predominantly C_2_^+^ and the contribution of C_4_^++^ is calculated using a peak decomposition algorithm by the help of the peak at 25.5 Da which corresponds to C_4_^++^ (^13^C^12^C_3_^++^) ^2,3^.


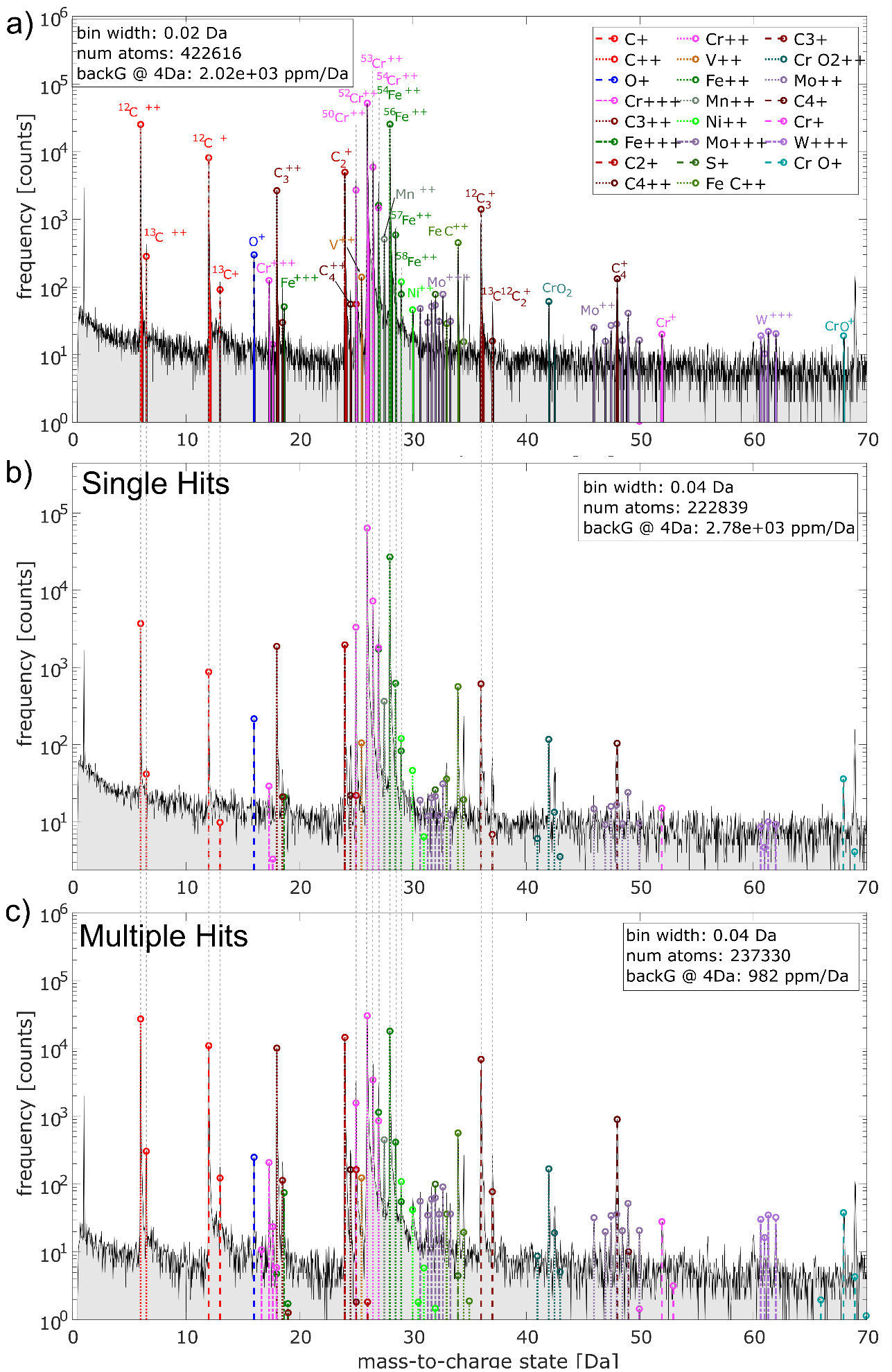


Figure S1: Mass spectrum of a carbide measured at 70ºK. a) Complete spectrum, b) Single hits, c) Multiple hits. C ions are relatively more frequent in multiple hits, but also the other elements have a high amount of multiple hit events. The ions that were analyzed for Miyamoto correction ^4^ are identified with grey lines and include the isotope ID.

Another possible source of error is the occurrence of preferential evaporation, since the ions with lower evaporation field (Fe and Cr respect to C) have more probability to evaporate between pulses and not be detected. This effect is more significant at high temperatures and lower pulse frequency ^5^. However, in the present analysis, the composition obtained at different temperatures does not show a significant variation, indicating a low influence of the previous effect in the measurement conditions.

Finally, the carbon quantification can also be affected by the pile-up effect in the detector due to the occurrence of multiple hit events. Analysis of the multiple and single events (table S1 and Figure S1) showed that C is mostly detected in multiple hits, having thus more probability of not being detected due to pile up effect on the detector ^3^. Also differences between measured and expected abundances of the most common isotopes are an indication of pile-up occurrence, since the higher the concentration, the higher the probability of pile-up ^6^. As can be seen in the carbide mass spectrum for all events and for multiple hit events (Figure S1), the most important peaks in the spectrum are C++, C+, Cr++, Fe++ and C_3_^+^. When analyzing the ratio between the most abundant isotope and the total detection for those ions, it is observed that the measured ratio is always lower than the natural abundance (Figure S2), indicating a detection loss occurrence. Given that, a correction for Fe++ pile-up proposed by Miyamoto et.al 2012 ^4^ was applied, and extended for C++, C+, Cr++ and C3+.

Table S1. Multiple and single hit events occurrence for relevant ions. Just isotopes that do not overlap are included. Total ranged counts inside the carbide: 358 375. T:70 ºK.

| **Ion** | **Single Hits** | **Multiple Hits** | **Ratio Multiple/Single** |
| --- | --- | --- | --- |
| ^12^C++ | 4278 | 30590 | 7.2 |
| ^13^C++ | 86 | 565 | 6.6 |
| ^12^C+ | 1306 | 14731 | 11.3 |
| ^13^C+ | 48 | 259 | 5.4 |
| ^52^Cr++ | 98232 | 56963 | 0.6 |
| ^53^Cr++ | 10442 | 10488 | 1.0 |
| ^56^Fe++ | 40016 | 29987 | 0.7 |
| ^57^Fe++ | 1028 | 1008 | 1.0 |


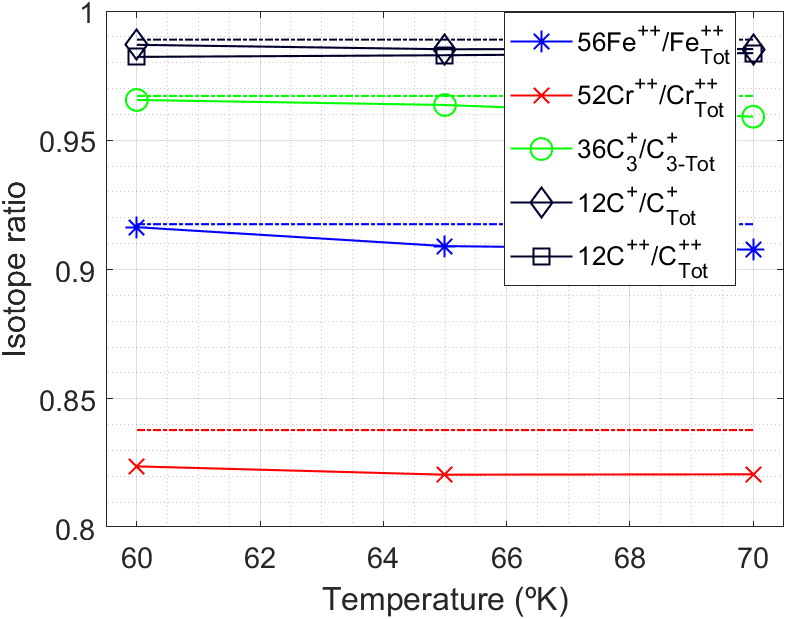


Figure S2: Isotope ratio for the most abundant ions measured inside the carbides. The solid line represent the measured ratio and the dashed line the natural abundance.

Miyamoto et.al 2012 ^4^ proposed that the detected events for the isotope i, $n_{Zi}$ can be estimated as:

$n_{Zi}=N.p.X_{Zi}-\alpha.N.p.{(X_{Zi})}^{2}$ [equation 1]

With $N$= number of evaporated Z ions, $p$= detector efficiency, $X_{Zi}$=natural abundance of the isotope and $\alpha$= parameter related to the possibility of multiple and close position evaporation. From that they derived the two following equations:

$\sum_{i} n_{Zi}=N.p.(1-\alpha.\sum_{i} \left( X_{Zi} \right)^{2})=A$ [equation 2]

$\sum_{i} n_{Zi}.X_{Zi}=N.p.(\sum_{i} \left( X_{Zi} \right)^{2}-\alpha.\sum_{i} \left( X_{Zi} \right)^{3})=B$ [equation 3]

Since A and B can be determined from natural abundances and the experimental count of each isotope (after background subtraction), the parameter $\alpha$ as well $N.p$ can be calculated.

$N.p$ represents the number of ions, accounting all the isotopes, that would be detected if no detection loss for pile-up occurs. $\alpha.\sum_{i} \left( X_{Zi} \right)^{2}$ corresponds to the detection loss.

Following this procedure, we corrected the total counts of C^++^(i= 12, 13), C^+^ (i=12, 13), C_3_^+^(i= 36, 37, 39), Cr^++^ (i= 50, 52, 53, 54) and Fe^++^ (i=54, 56, 57, 58) and we recalculated the carbide compositions. The natural abundances were extracted from IVAS^TM^ 3.6.14 (CAMECA Instruments, Madison, Wi, USA).

The uncorrected and corrected C, Fe and Cr compositions as well as the % of detection loss are presented in figure S3. Moreover, the quantification of each individual element, measured at 3 different temperatures, is shown in the table below.


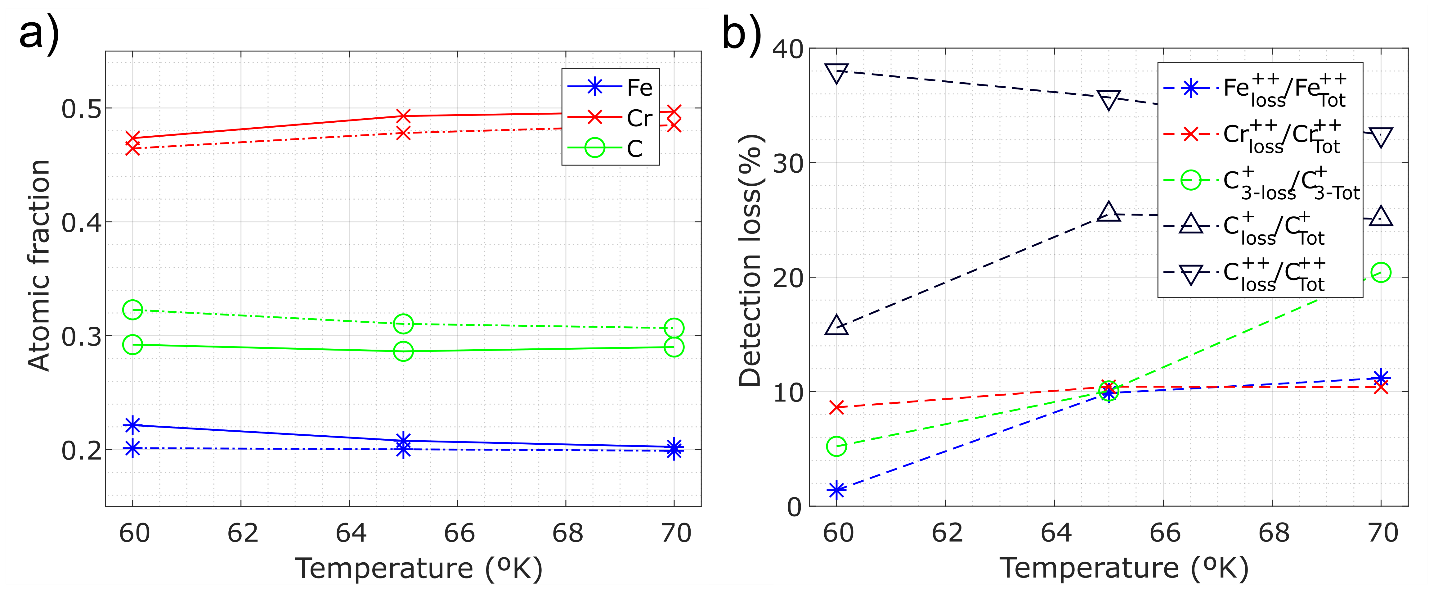


Figure S3: a) Fe, Cr and C content inside the carbides measured at different temperatures. The solid line correspond to the result obtained after peak decomposition while the doted line represent the composition after applying the correction suggested by Miyamoto et.al. 2012 ^4^. b) % of ions non detected with respect to the total ions that would be detected if pile-up does not occur for the same ion ^4^. As expected, the C ions that have a high occurrence of multiple events are relatively more affected by the pile-up phenomenon. Ion number in the analysis: 60ºK: 776.942, 65ºK: 1.107.086, 70ºK: 388.257.

Table S2: Chemical elements quantification measured at three different temperatures

|  | **Composition (at%)** | | | | | |
| --- | --- | --- | --- | --- | --- | --- |
| **T(ºK)** | **60** | | **65** | | **70** | |
|  | Decomposed | Corrected | Decomposed | Corrected | Decomposed | Corrected |
| **C** | 29.2% | 32.3% | 28.6% | 31.0% | 29.0% | 30.7% |
| **Cr** | 47.4% | 46.4% | 49.3% | 47.8% | 49.7% | 48.5% |
| **Fe** | 22.2% | 20.1% | 20.8% | 20.0% | 20.2% | 19.9% |
| **Mn** | 0.601% | 0.54% | 0.57% | 0.50% | 0.37% | 0.32% |
| **Mo** | 0.372% | 0.33% | 0.39% | 0.342% | 0.342% | 0.30% |
| **Ni** | 0.004% | 0.003% | 0.008% | 0.007% | 0.050% | 0.04% |
| **S** | 0.011% | 0.01% | 0.001% | 0.001% | 0.017% | 0.01% |
| **Si** | 0.016% | 0.014% | 0.007% | 0.006% | 0.011% | 0.01% |
| **V** | 0.20% | 0.18% | 0.24% | 0.21% | 0.22% | 0.19% |
| **W** | 0.067% | 0.060% | 0.065% | 0.056% | 0.064% | 0.056% |

From those values it was determined that the final C content of the carbides is in average 31±1 at.%. From this analysis we can confirm that the carbides precipitating close to the EC corresponds to M_7_C_3_. We also observed that the most favorable measurement temperature is 70ºK, which gives a composition closer to the stoichiometric (30.7 at. %).

**References**

1. Vurpillot, F., Bostel, A. & Blavette, D. Trajectory overlaps and local magnification in three-dimensional atom probe. *Appl. Phys. Lett.* **76**, 3127–3129 (2000).

2. Takahashi, J., Kawakami, K. & Kobayashi, Y. Quantitative analysis of carbon content in cementite in steel by atom probe tomography. *Ultramicroscopy* **111**, 1233–1238 (2011).

3. Marceau, R. K. W., Choi, P. & Raabe, D. Understanding the detection of carbon in austenitic high-Mn steel using atom probe tomography. *Ultramicroscopy* **132**, 239–247 (2013).

4. Miyamoto, G., Shinbo, K. & Furuhara, T. Quantitative measurement of carbon content in Fe-C binary alloys by atom probe tomography. *Scr. Mater.* **67**, 999–1002 (2012).

5. Blavette, D., Déconihout, B., Chambreland, S. & Bostel, A. Three-dimensional imaging of chemical order with the tomographic atom- probe. *Ultramicroscopy* **70**, 115–124 (1998).

6. Takahashi, J., Kawakami, K. & Raabe, D. Comparison of the quantitative analysis performance between pulsed voltage atom probe and pulsed laser atom probe. *Ultramicroscopy* **175**, 105–110 (2017).
